# Sclerostin is upregulated in the early stage of chondrogenic differentiation, but not required in endochondral ossification *in vitro*

**DOI:** 10.1371/journal.pone.0201839

**Published:** 2018-08-02

**Authors:** Yasuteru Yamaguchi, Ken Kumagai, Sosuke Imai, Kazuma Miyatake, Tomoyuki Saito

**Affiliations:** Department of Orthopaedic Surgery and Muscloskeletal Science, Graduate School of Medicine, Yokohama City University, Yokohama, Japan; Kyungpook National University School of Medicine, REPUBLIC OF KOREA

## Abstract

Sclerostin is a potent inhibitor of the canonical Wnt signaling pathway. Wnt signaling pathways have multiple roles in the regulation of cartilage development, growth, and maintenance. This study focused on the role of sclerostin in the process of chondrogenic differentiation. We hypothesized that sclerostin is essential to induce chondrogenic differentiation and regulate endochondral ossification. ATDC5 cells were used to investigate chondrogenic differentiation and terminal calcification. During chondrogenic differentiation, intrinsic sclerostin was upregulated in the early stage, but downregulated in the late stage. Addition of sclerostin elevated expressions of Sox9 and Col2a1 (P<0.05) and reduced expressions of Runx2, Col10a1, MMP-3, MMP-13, and ADAMTS5 (P<0.05) through inhibition of the Wnt-β-catenin signaling pathway (P<0.05). Terminal calcification was significantly inhibited by sclerostin (P<0.05). In contrast, deletion of sclerostin decreased expressions of Sox9 and Col2a1 (P<0.05), increased expressions of Runx2, Col10a1, MMP-3, and MMP-13 (P<0.05), and promoted terminal calcification (P<0.05). This study provides insights into the possible role of sclerostin in the regulation of chondrogenic differentiation. Sclerostin is upregulated in the early stage of chondrogenic differentiation, but is not required in endochondral ossification. Sclerostin is a candidate modulator for chondrogenic differentiation.

## Introduction

Chondrocyte differentiation plays an important role in longitudinal skeletal development that terminates with endochondral ossification. The process of endochondral ossification begins by forming a cartilage template and ends with its replacement by bone. Sequential differentiation of chondrocytes is highly regulated by transcription factors, extracellular matrix proteins, and several signaling pathways including Wnt signaling [1]. Wnt signaling regulates the fate of mesenchymal stem cells to differentiate into either chondrocyte or osteoblast in the skeletal development [2]. Differentiation toward chondrogenic lineage is initially induced by low levels of β-catenin-dependent canonical wnt signaling, which promotes Sox9 expression and activity [3]. In adult articular chondrocyte, the phenotype is maintained with a well-balanced β-catenin level [3]. In contrast, chondrogenic hypertrophy is induced by high levels of wnt/β-catenin signaling [2–4].

Sclerostin is known to be a negative regulator of bone formation, expressed by the SOST gene in osteocytes. SOST is a ligand for LRP5/LRP6 and a potent inhibitor of the canonical Wnt signaling pathway [5, 6]. Recent studies have shown that the SOST gene is also expressed by chondrocytes [7], and that modulation of its activity may have effects on articular cartilage and subchondral bone [8]. Extensive studies of the function of sclerostin may lead to understanding the mechanisms of bone and cartilage physiology and pathology. However, the role of sclerostin in the progression of chondrogenic differentiation has not yet been well elucidated, and the potential utility of sclerostin for the regulation of endochondral ossification is not known.

This study focused on the role of sclerostin in the process of chondrogenic differentiation. We hypothesized that sclerostin is essential to induce chondrogenic differentiation and regulate endochondral ossification. The sclerostin expression level during chondrogenic differentiation *in vitro* was investigated, and whether modulation of SOST expression controls chondrocyte differentiation was examined.

## Materials and methods

### Immunohistochemistry for sclerostin

Three C57BL/6 mice were purchased from the Japan SLC Inc. (Hamamatsu, Japan) and used in this study. The animals were housed in cages in ventilated racks under a 12-h light/dark cycle with unrestricted access to food and water. Animals were euthanized at 15 weeks old by inhalation of carbon dioxide gas. Knee joints obtained from mice were fixed in 4% paraformaldehyde, decalcified in an EDTA-based decalcifying solution, dehydrated in an ascending series of ethanol, embedded in paraffin, and cut into 5-μm-thick sections. The tissue sections were deparaffinized and rehydrated. Endogenous peroxidase was quenched for 10 minutes with 3% H_2_O_2_ in water. Nonspecific binding was blocked with 3% bovine serum albumin in phosphate-buffered saline (PBS). Anti-sclerostin antibody (AF1589, R&D systems, Minneapolis, MN, USA) was incubated overnight at 4°C. The next day, slides were washed in PBS and incubated with biotinylated anti-rabbit IgG antibody for 45 minutes at room temperature. Normal rabbit IgG was used for negative control. The reaction was visualized by incubation with the avidin-biotin-peroxidase reagent included in the Vectastain ABC Kit (Vector Laboratories, Burlingame, CA, USA), followed by color development with 3’3-diaminobenzidine tetrahydrochloride (Dojindo, Kumamoto, Japan). Finally, the sections were counterstained with hematoxylin and mounted with coverslips. All animal protocols were approved by Institutional Animal Care and Use Committees at Yokohama City University (Protocol Number: F-A-17-088).

### Cell lines and culture conditions

For chondrogenic induction, ATDC5 cells were cultured in a 1:1 mixture of Dulbecco’s modified Eagle’s (DME) and Ham’s F12 (DME/F12) medium (Flow Laboratories, Irvine, UK) containing 5% fetal bovine serum (FBS: GIBCO, New York, NY, USA), 10 μg/ml bovine insulin (I; Wako Pure Chemical, Osaka, Japan), 10 μg/ml human transferrin (T; Boehringer Mannheim, Mannheim, Germany), and 3 x 10^−8^ M sodium selenite (S; Sigma Chemical Co., St. Louis, MO, USA) at 37°C in a humidified atmosphere of 5% CO_2_ in air for the initial 3 weeks, as previously described [9]. The inoculum density of the cells was 2 x 10^4^ cells/well in a 24-multiwell plate, 4 x 10^4^ cells/well in a 12-multiwell plate, or 6 x 10^4^ cells/well in a 6-multiwell plate (Corning, New York, NY, USA). On day 21, the culture medium was switched to alpha modified essential medium (α-MEM) containing 5% FBS plus ITS, and the CO_2_ concentration was shifted to 3% to facilitate mineralization in culture. The medium was replaced every other day. The effect of sclerostin was examined by addition of 20 ng/ml recombinant mouse SOST (R&D systems).

### Lentiviral-mediated shRNA gene silencing

ATDC5 cells were transduced with lentivirus-mediated shRNA nonspecific control (sc-108080) or lentivirus-mediated shRNA targeting SOST (shSOST; sc-61504-V) from Santa Cruz Biotechnology (Santa Cruz, CA, USA). Briefly, 2 × 10^5^ cells were transduced with lentiviral by spinoculation at multiplicity of infection equal to 3 and selected by puromycin (sc-108071, 2.0 μg/mL). The medium was replaced with fresh puromycin-containing medium every 3–4 days, until resistant colonies could be identified. Resistant colonies were collected, and control shRNA lentiviral particles and sclerostin shRNA lentiviral particles were compared.

### Alcian blue staining

To evaluate the deposition of sulfated glycosaminoglycan (sGAG) during chondrogenic differentiation, proteoglycan-rich matrix was stained with Alcian blue. Cells were fixed with 100% methanol and stained with 0.1% Alcian blue 8GS (Sigma) in 0.1N HCl for 4 h at room temperature.

### Alizarin red staining

Cells were fixed with phosphate-buffered formalin and then stained with 40 mM alizarin red S (pH 4.2, Sigma) for 30 min. After washing the wells with pure water, the plates were photographed. Alizarin red dye was extracted with 10% formic acid, and the absorbance at 450 nm was determined with a microplate reader (Infinite F50, TECAN, Kawasaki, Japan).

### sGAG assay

Quantification of sGAG in the media was performed using a commercial sGAG alcian blue binding assay kit (Euro-Diagnostica, Malmö, Sweden). The absorbance at 640 nm was measured using a microplate reader (Infinite F50, TECAN).

### Total RNA isolation and real-time RT-PCR

Total RNA was isolated using Trizol reagent (Invitrogen, Carlsbad, CA, USA). RNA was quantified by measuring absorbance at 260 nm, and the quality was assessed by determining the 260/280 nm absorbance ratio. First-strand cDNA synthesis was performed with 0.5 μg or 1 μg of total RNA in a total volume of 20 μl using an iScriptTM advanced cDNA synthesis kit (BIO-RAD, Richmond, CA, USA). Quantitative real-time PCR was carried out using TaqMan gene expression assays (Applied Biosystems, Foster City, CA, USA) on a CFX96TM real-time PCR detection system (BIO-RAD) in a 20-μl reaction volume. Expression of the gene of interest was normalized to GAPDH expression. TaqMan gene expression assays used in this study were as follows: SOST (Mm00470479_m1); Col2a1 (Mm01309565_m1); Col10a1 (Mm00487041_m1); Sox9 (Mm00448840_m1); Runx2 (Mm00501584_m1); Wnt3a (Mm03053669_s1); Wnt5a (Mm00437347_m1); LRP5 (Mm01227476_m1); LRP6 (Mm00999795_m1); Axin2 (Mm00443610_m1); Ctnnbl1 (β-catenin) (Mm00499427_m1); MMP3 (Mm00440295_m1); MMP13 (Mm00439491_m1); ADAMTS5 (Mm00478620_m1); and GAPDH (Mm99999915_g1).

### Statistical analysis

All experiments were repeated independently at least three times. All data are presented as means ± SEM. The analysis was done using SigmaStat 3.5 software (Systat Software Inc., Richmond, CA, USA). The non-parametric Kruskal-Wallis test was used to test for significant differences among the test groups. When a significant difference was detected, Steel’s post hoc test was performed to compare each of the treatments with a control. An adjusted P value < 0.05 was considered significant.

## Results

### Expression of sclerostin in chondrogenic differentiation

**Fig 1:**
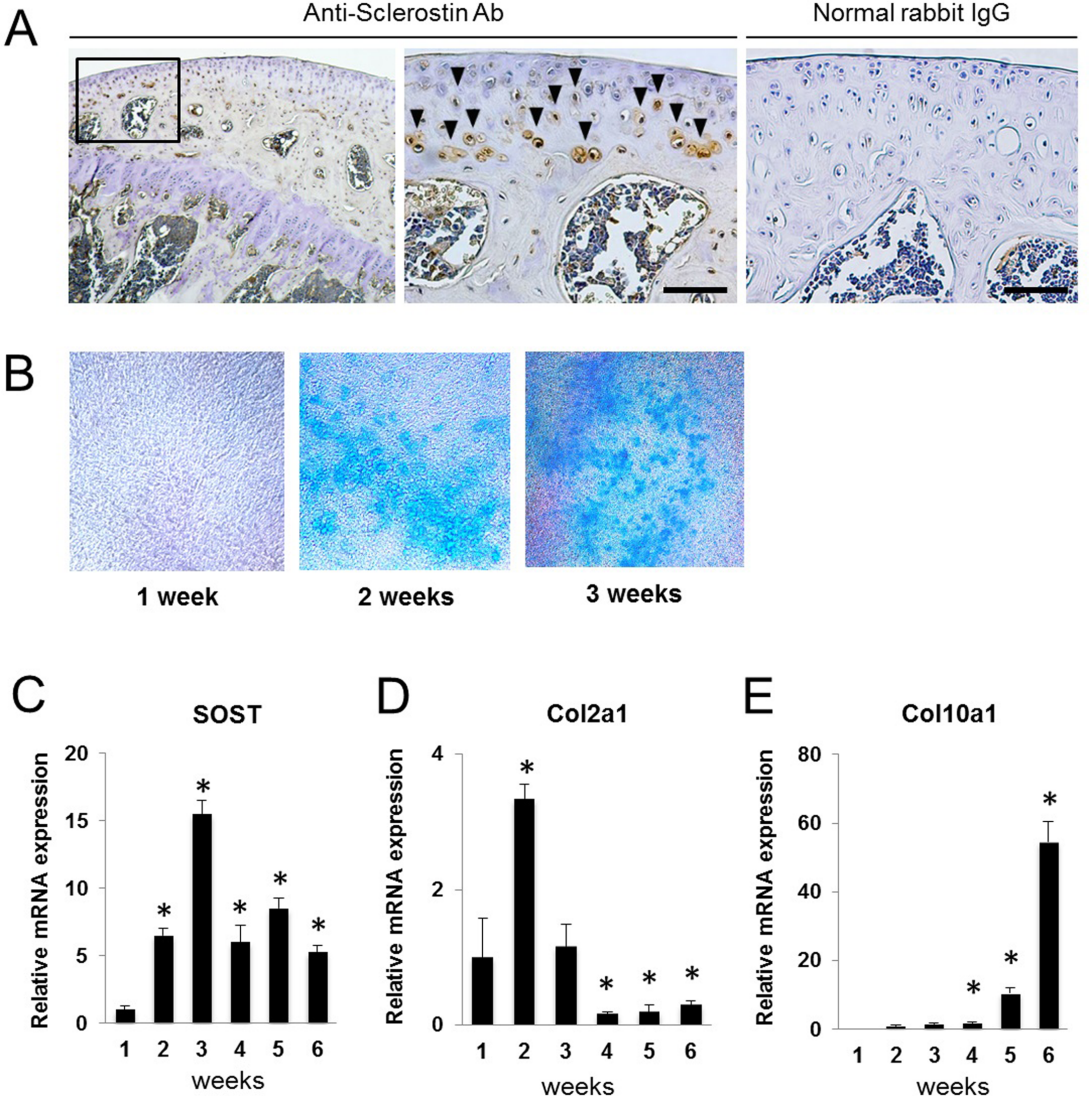
Relationship between chondrogenic differentiation and intrinsic expression of sclerostin. (A) Immunostaining of sclerostin in mouse knee joints. Sclerostin-positive cells are labeled with brown in the deep layer of articular cartilage (arrowheads). Note that no background staining is observed in the section incubated with normal rabbit IgG. Scale bar = 50 μm. (B) Alcian blue staining of ATDC5 cells under chondrogenic culture for 3 weeks. (C-E) Relative mRNA expressions of SOST (C), Col2a1 (D), and Col10a1 (E) during chondrogenic differentiation of ATDC5 cells for 6 weeks. N = 4, *P<0.05 vs 1 week.

Immunohistochemical findings of the mouse knee joints showed that sclerostin-positive cells were localized in the deep layer of articular cartilage (Fig 1A). Chondrogenic differentiation of ATDC5 cells was confirmed by positive staining with Alcian blue after 2 weeks (Fig 1B). To investigate the relationship between sclerostin and the chondrogenic differentiation process of ATDC5 cells, mRNA expressions of SOST, COL2A1 (chondrogenic differentiation marker), and COL10A1 (hypertrophic chondrocyte marker) for 6 weeks of culture were investigated by real-time RT-PCR (Fig 1C). SOST was identified after 1 week of culture. Then, the expression level increased and peaked at 3 weeks. Corresponding with SOST expression, Col2a1 was elevated up to two weeks. After 4 weeks, expression of SOST was decreased, and Col10a1 was upregulated. Thus, the expression level of SOST was altered during the chondrogenic differentiation process.

### Effect of sclerostin on the early stage of chondrogenic differentiation

**Fig 2:**
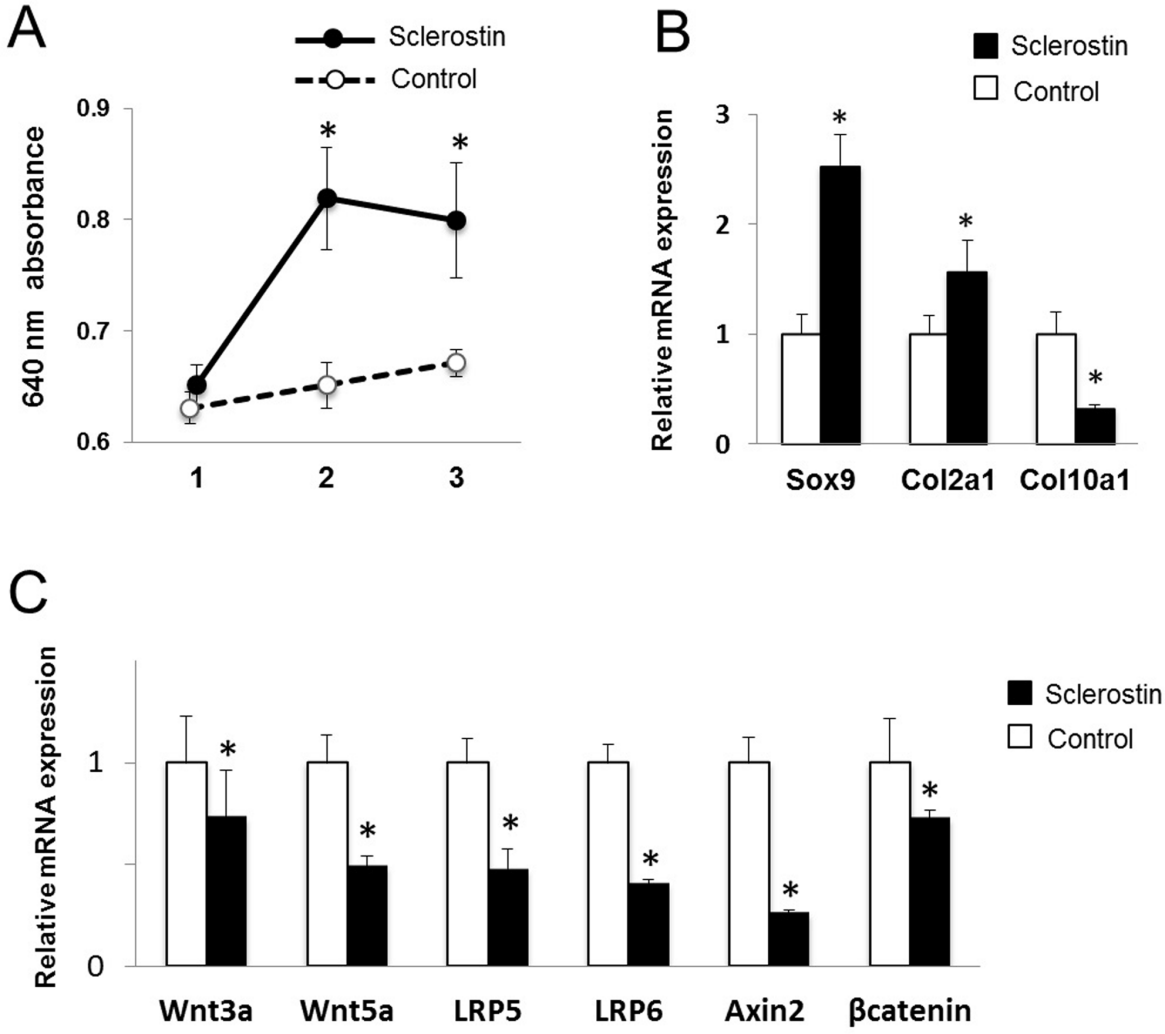
Effects of sclerostin on the early stage of chondrocyte differentiation. ATDC5 cells were cultured under chondrogenic conditions, and 20 ng/ml sclerostin were added to the culture medium. (A) Absorbance at 640 nm of sGAG in the culture solution. N = 4, *P<0.05 vs control. (B) Relative mRNA expressions of Sox9, Col2a1, and Col10a1 at 3 weeks of culture. N = 4, *P<0.05 vs control. (C) Relative mRNA expressions of Wnt3a, Wnt5a, LRP5, LRP6, Axin2, and β-catenin at 3 weeks of culture. N = 4, *P<0.05 vs control.

Sclerostin was added to culture medium, and chondrogenic differentiation and proteoglycan synthesis were assessed. The GAG concentration in the culture supernatant was significantly elevated by addition of sclerostin (Fig 2A). Expressions of Sox9 and Col2a1, markers for the early stage of chondrogenic differentiation, were significantly elevated by addition of sclerostin, while expression of Col10a1, a marker for late-stage differentiation, was significantly decreased (Fig 2B). Expressions of Wnt/β**-**catenin target genes, Wnt3a, Wnt5a, LRP5, LRP6, Axin2, and β**-**catenin were significantly reduced by addition of sclerostin (Fig 2C). The results suggested that sclerostin promotes differentiation of proliferating chondrocytes by inhibiting Wnt/β-catenin signaling in the early stage of chondrogenic differentiation.

### Effect of sclerostin on the late stage of chondrogenic differentiation

**Fig 3:**
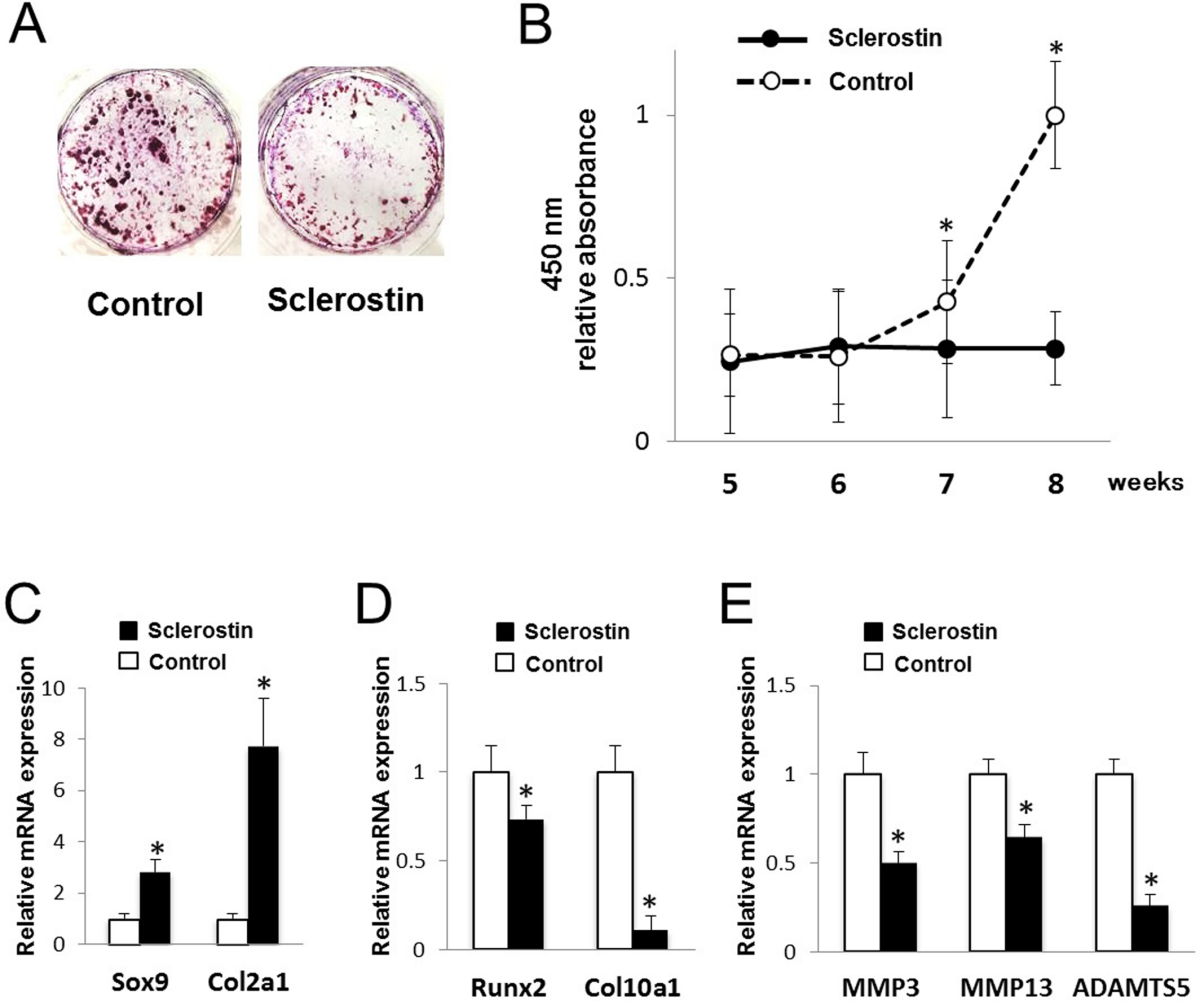
Effect of sclerostin on the late stage of chondrocyte differentiation. ATDC5 cells were cultured for endochondral ossification, and 20 ng/ml sclerostin were added to the culture medium from the 21st day of culture. (A) Alizarin red staining after 8 weeks. (B) Relative absorbance at 450 nm of calcified eluate from alizarin red staining. N = 4, *P<0.05 vs control. (C-E) Relative mRNA expressions of Sox9 and Col2a1 (C), Runx2 and Col10a1 (D), and MMP3, MMP13, and ADAMTS5 (E) at 6 weeks. N = 4, *P<0.05 vs control.

To assess the effect of sclerostin on the late stage of chondrogenic differentiation, sclerostin was added from 3 weeks of culture. Terminal calcification was significantly inhibited by sclerostin (Fig 3A and 3B). Expressions of Sox9 and Col2a1 were significantly increased by sclerostin, whereas expressions of Runx2 and Col10a1 were significantly decreased by sclerostin (Fig 3C). Furthermore, expressions of MMP-3, MMP13, and ADAMTS5, catabolic chondrocyte markers, were significantly reduced by sclerostin (Fig 3D). Thus, the results suggest that sclerostin inhibited late-stage chondrogenic differentiation.

### Effects of sclerostin knockdown on chondrogenic differentiation

**Fig 4:**
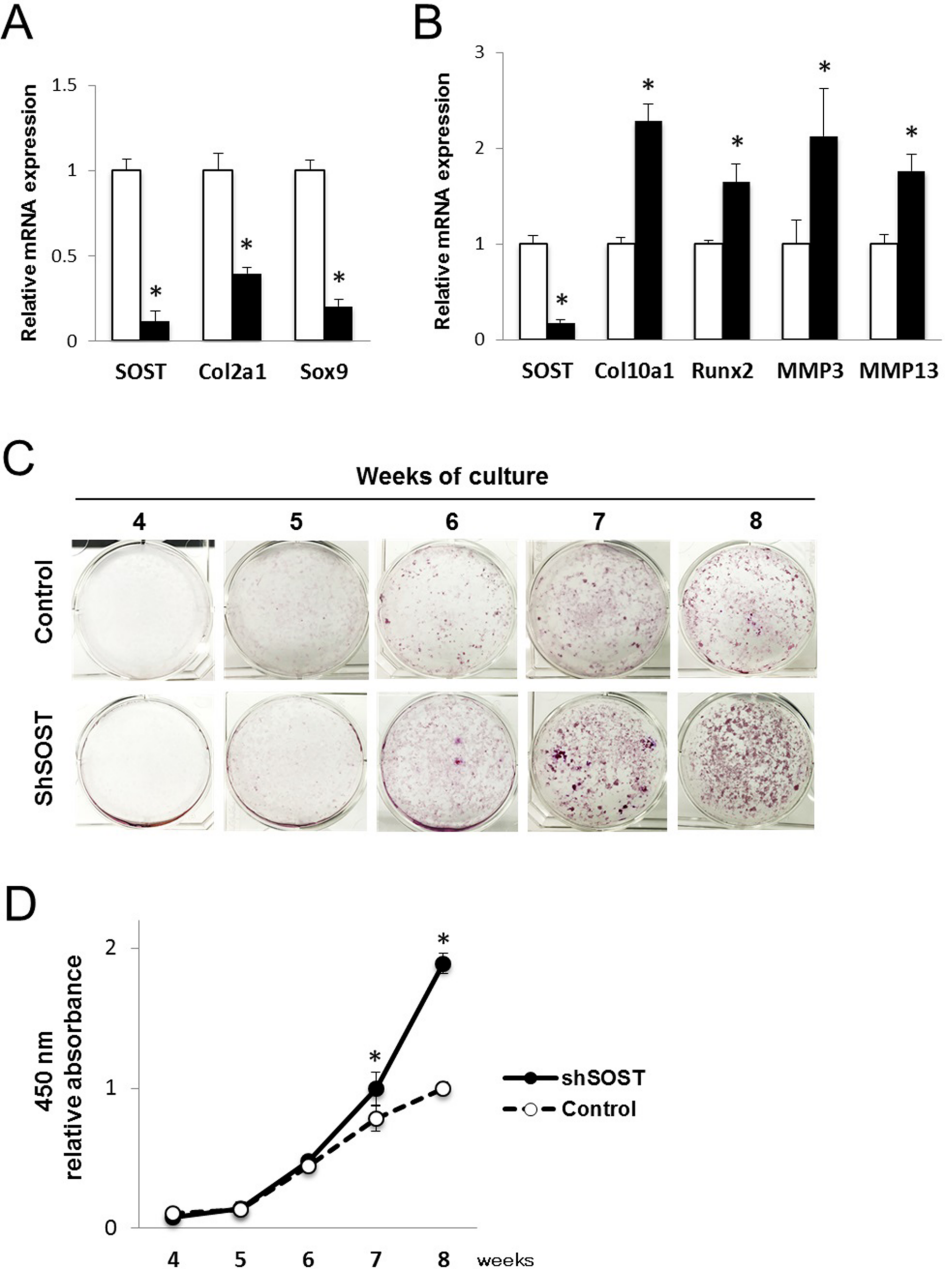
Sclerostin silencing promotes endochondral ossification. ATDC5 cells with SOST gene silencing (shSOST) were cultured for endochondral ossification. (A) Relative mRNA expressions of SOST, Col2a1, and Sox9 at 2 weeks of culture. N = 4, *P<0.05 vs control. (B) Relative mRNA expressions of SOST, Col10a1, Runx2, MMP3, and MMP13 at 5 weeks of culture. N = 4, *P<0.05 vs control. (C) Alizarin red staining after 4 to 8 weeks. (D) Relative absorbance at 450 nm of calcified eluate from alizarin red staining. N = 4, *P<0.05 vs control.

To further assess whether deletion of sclerostin promotes terminal differentiation of chondrocytes, chondrogenic differentiation was examined under the condition of SOST knockdown. Expressions of SOX9 and Col2a1 at 2 weeks of culture were decreased in association with attenuation of the SOST gene (Fig 4A). In contrast, expressions of Col10a1, Runx2, MMP-3, and MMP-13 at 5 weeks of culture were significantly increased by reduction of the SOST gene (Fig 4B). Terminal differentiation of chondrocytes was assessed by detection of calcium deposits in ATDC5 cells with or without the SOST gene. When comparing the same time points of culture, the SOST knockdown group showed greater calcified nodule formation than the control group (Fig 4C). These findings showed that deletion of the SOST gene in the late stage accelerates terminal differentiation.

## Discussion

The present study demonstrated that sclerostin is expressed in chondrocytes and upregulates the early stage of chondrogenic differentiation *in vitro*. Furthermore, sclerostin downregulates the expression of markers associated with the late stage of chondrogenic differentiation. Thus, induction of chondrogenic differentiation and restoration of the chondrogenic phenotype may be dependent on the presence of sclerostin. In contrast, the late stage of chondrogenic differentiation is inhibited by sclerostin. It is most likely that sclerostin is not required in terminal chondrogenic differentiation toward endochondral ossification.

As described in detail previously [10], ATDC5 is a good model system for studying the dynamic processes of chondrogenesis, and many findings in this system may have relevance to chondrogenesis *in vivo*. The present study focused on sclerostin as an inhibitor of the canonical Wnt signaling pathway in the multistep differentiation process encompassing the stages from mesenchymal condensation to calcification *in vitro* [11]. As described in detail previously [4], β-catenin-dependent canonical Wnt signaling is required for progression of endochondral ossification and growth of axial and appendicular skeletons, while excessive activation of this signaling can cause severe inhibition of initial cartilage formation and growth plate organization and function. Increased canonical Wnt signaling inhibits chondrogenesis [12, 13], but once cartilage has formed, it promotes chondrocyte maturation, enhances perichondral bone formation, initiates cartilage vascularization, and drives the formation of primary and secondary ossification centers [14]. The present study supports these previous reports through regulation of sclerostin. Namely, downregulation of Wnt signaling by increased expression of SOST promotes proliferative chondrocytes in the early stage, and upregulation of Wnt signaling by reduced expression of SOST induces terminal differentiation toward endochondral ossification in the late stage.

Sclerostin is commonly referred to as an osteocyte-specific protein and clinically focused as an emerging therapeutic target for treating osteoporosis and osteoporotic fracture [8, 15]. In addition, the recent findings of sclerostin expression in chondrocytes shed light on the potential contribution to the pathogenesis of cartilage diseases, including osteoarthritis (OA) [16]. OA is characterized by cartilage degradation and osteophyte formation. Signals for endochondral ossification, including chondrocyte hypertrophy and cartilage apoptosis, play a key role in the progression of OA [17]. Wnt/β-catenin signaling could stimulate cartilage matrix degradation mediated by proteases including matrix metalloproteinases and aggrecanases in chondrocytes [18, 19]. Thus, inhibition of Wnt/β-catenin signaling by sclerostin seems to be a potential treatment target for OA. The present study confirmed that sclerostin downregulates expressions of markers for chondrocyte hypertrophy and matrix catabolism and prevents endochondral ossification. Sclerostin regulates disease processes in OA by opposing the effects of promoting disease-associated subchondral bone sclerosis, while inhibiting degradation of cartilage [20]. Sclerostin may contribute to maintenance of cartilage integrity in OA [21]. It remains to be elucidated whether interventions that alter sclerostin expression have potential utility for treating OA. Furthermore, endochondral ossification within bone healing was delayed in the mice fracture model in which the Wnt/β-catenin signaling is specially inhibited in chondrocytes [22]. Sclerostin may potentially have negative effects on fracture healing under the process of endochondral ossification.

This study provides insights into the possible role of sclerostin in the regulation of chondrogenic differentiation through the dynamic processes encompassing the stages from the initial step to the terminal calcification *in vitro*. Sclerostin is upregulated in the early stage of chondrogenic differentiation, but it is not required in endochondral ossification. Thus, sclerostin may contribute positively to the cartilage maintenance and negatively to the terminal calcification. Sclerostin is a candidate modulator for chondrogenic differentiation.
